# “It just isn’t the same”: altered routines among older Americans three years after the COVID-19 pandemic onset

**DOI:** 10.3389/fpubh.2025.1573302

**Published:** 2025-07-09

**Authors:** Rebecca A. Milan, Mallory A. P. Sagehorn, Rohini Perera, Grace I. Bowman, Jessica Finlay

**Affiliations:** ^1^Department of Epidemiology, Center for Social Epidemiology and Population Health, University of Michigan School of Public Health, Ann Arbor, MI, United States; ^2^Department of Geography, University of Colorado Boulder, Boulder, CO, United States; ^3^Institute of Behavioral Science, University of Colorado Boulder, Boulder, CO, United States; ^4^Department of Epidemiology, University of Michigan School of Public Health, Ann Arbor, MI, United States; ^5^Survey Research Center, University of Michigan Institute for Social Research, Ann Arbor, MI, United States; ^6^Department of Ecology and Evolutionary Biology, University of Colorado Boulder, Boulder, CO, United States; ^7^Institute for Behavioral Genetics, University of Colorado Boulder, Boulder, CO, United States; ^8^Biofrontiers Institute, University of Colorado Boulder, Boulder, CO, United States

**Keywords:** older adults, COVID-19, routines, pandemic, aging

## Abstract

**Introduction:**

The COVID-19 pandemic significantly disrupted civic life, particularly for older adults at increased risk for severe morbidity and mortality. Yet, little is known about the longer-term impacts on their daily routines and how this may affect health and wellbeing.

**Methods:**

This qualitative study utilized data from older US adults who participated in the COVID-19 Coping Study’s three-year follow-up online survey, conducted in April–May 2023. The primary aim was to understand *how* and *why* daily routines have changed among older Americans (*N* = 1,309).

**Results:**

Participants had an average age of 71 years, with approximately 74% female and 93% identifying as Non-Hispanic White. We conducted content and thematic analysis of open-ended survey responses to identify five key reasons for still-altered routines 3 years after the pandemic onset: (1) COVID-19 risk and exposure, (2) altered access, (3) broader life circumstances, (4) emotional health, and (5) physical health.

**Discussion:**

These findings highlight the enduring impacts of the pandemic on older adults’ routines and underscore the importance of integrating public health strategies that prioritize routine stability to enhance mental, physical, and social health. To support older adults’ wellbeing during and beyond public health emergencies, we recommend strengthening community-based programs, improving access to health and social services, and designing adaptable interventions that help individuals rebuild and maintain meaningful daily routines.

## Introduction

The COVID-19 pandemic significantly disrupted daily routines for older adults, resulting in profound changes to social interactions, mobility, and access to essential services. Daily routines, regular, repeated patterns of behavior that structure daily life, are closely tied to health and wellbeing in later life (1, 2). Disruption to these routines during the pandemic led to restricted mobility and limited access to key amenities and “third places” (3) such as restaurants, recreation centers, and places of worship (4), thereby heightening risk for social isolation, reduced physical activity, and limited opportunities for cognitive stimulation (5–7). Although many services shifted to online platforms and home-based alternatives, these adaptations had uneven impacts on older adults depending on digital access, ability, and comfort (8). While some older adults demonstrated resilience by adapting to the “new normal” and finding ways to stay active (9, 10), the long-term implications of these disruptions remain unclear. This is a significant gap, as older adults are particularly vulnerable to the downstream effects of sustained changes in routines, which may influence physical, mental, and cognitive health through behavioral and social pathways.

The magnitude of this issue is underscored by evidence linking pandemic-related disruptions to declines in older adults’ mental health, increased social stress, and reduced community engagement due to fears of exposure (11). Moreover, social isolation and loneliness, both of which intensified during the pandemic, are well-documented risk factors for adverse health outcomes, including depression, dementia, and cardiovascular disease (12). The closure of recreational and social facilities, loss of in-person networks, and limited access to cognitively stimulating environments compounded these risks (5, 13). While the initial disruptions are widely studied, the longer-term effect on overall wellbeing–particularly in a post-acute pandemic, endemic phase–are less understood (14, 15).

Emerging evidence suggests that many older adults continue to experience hesitation about reengaging in community spaces and report enduring changes in their routines and relationships with place (9). In some cases, community spaces no longer evoke the same sense of safety and belonging they once had (16). These trends raise concerns about prolonged disconnection from health-promoting environments.

This study addresses this critical gap by examining how and why older adults’ daily routines remain altered more than 3 years after the onset of the COVID-19 pandemic. By analyzing open-ended responses from participants in the COVID-19 Coping Study’s 2023 follow-up survey, we explore sustained impacts on routines and perceived barriers to returning to pre-pandemic life. The practical aim of this research is to inform public health strategies that promote routine stability, rebuild community connections, and ensure access to supportive resources during and beyond public health emergencies.

## Methods

The COVID-19 Coping Study is an online, longitudinal cohort study of older adults residing within the United States during and after the COVID-19 pandemic onset. The baseline survey was administered from April to May 2020 and recruited adults aged 55 years or old via a nonprobability sampling strategy (i.e., selective sampling and targeted outreach through professional, community, and retirement networks) due to the limited resources available at that time of the pandemic. Online survey administration was adopted at baseline due to pandemic-related restrictions and was maintained for the study’s duration. Participants completed a 20-min survey assessing health, wellbeing, and lifestyle changes during the early pandemic. The study’s overarching aim was to document short-and long-term effects of the pandemic on aging populations in the US and outlying US territories.

Following baseline data collection in 2020, participants were invited to complete annual surveys. This analysis uses data from the Year 3 follow-up survey, administered online between April and May 2023. Participants were recontacted via email using addresses provided at baseline. The response format was an online survey that included both closed-and open-ended questions. The retention rate from baseline to three-year follow-up was approximately 43.5%. The study received approval from the University of Michigan’s Institutional Review Board (HUM00179632) and all participants provided informed consent. Further details on study methodology, participant recruitment, inclusion criteria, and retention procedures have been published elsewhere (17, 18).

Sociodemographic information presented in Table 1 was collected at baseline in Spring 2020. In the Year 3 follow-up, participants were asked if they had moved since baseline, but all other demographic variables reflect initial responses.

**Table 1:** Sociodemographic of qualitative respondents, COVID-19 coping study, United States, 2023 (*N* = 1,309).

Characteristic	*n* (%)
Mean (SD) age	71 (7.0)
Sex	
Male	342 (26.1)
Female	964 (73.6)
Other	1 (0.1)
Prefer not to say	2 (0.2)
Race/ethnicity	
Non-Hispanic White	1,211 (92.5)
Non-Hispanic Black	33 (2.5)
Hispanic or Latinx	22 (1.7)
East Asian/Native Hawaiian/Other Pacific Islander	12 (0.9)
Asian Indian	2 (0.2)
American Indian or Alaska Native	9 (0.7)
Other	20 (1.5)
Education	
High school diploma or equivalent	19 (1.5)
Some college	140 (10.7)
College graduate	389 (29.7)
Graduate school	761 (58.1)
Employment status	
Homemaker	24 (1.6)
Working	376 (28.8)
Retired	851 (65.2)
Not working/in school/other	57 (4.4)
Marital status	
Married or in a relationship	857 (65.5)
Single, divorced/separated	185 (14.1)
Single, never married	114 (8.7)
Single, widowed	142 (10.8)
Other	11 (0.8)
Baseline US region of residence	
West	213 (16.3)
South	181 (13.8)
Midwest	773 (59.1)
Northeast	140 (10.7)
Outlying US territories	1 (0.1)
Baseline residence	
Urban	1,191 (91.1)
Rural	116 (8.9)
Moved since baseline	
No	1,106 (84.6)
Yes	201 (15.4)

SD, standard deviation. All characteristics were measured in April/May 2023, except for sex, race/ethnicity, education, marital status, and region of residence, which was measured in April/May 2020. All measures are unweighted.

The Year 3 follow-up survey included questions about participants’ current health, wellbeing, and lifestyle. Participants first answered yes/no questions about whether key routines had changed since the pandemic began, followed by open-ended prompts on *how* and *why* their routines were different (Appendix A). Open-ended questions focused on changes to social, exercise, and creative/educational activity routines based on their importance to health and wellbeing in later life (19). Participants’ qualitative responses ranged from a few words to several sentences. For the purpose of this analysis, we included participants who responded to at least one of the three qualitative routine responses.

Although the broader study includes both quantitative and qualitative components, this manuscript presents a qualitative-only analysis. We intentionally focus on open-ended responses to better understand the nuanced ways in which older adults experienced and adapted their routines in the years following the pandemic’s onset. This approach aligns with Brower et al. (20) “Big Qual” framework, in which large-scale qualitative data across geographically and demographically varied populations supports rich interpretive analysis of shared and divergent experiences (20).

We used a combined approach of content analysis and thematic analysis to identify patterns in how and why routines changed. Given the large volume of qualitative data, content analysis provided a useful foundation for identifying recurring concepts, which in turn informed the development of more nuanced themes. To guide our interpretation of how older adults experienced routine change throughout the COVID-19 pandemic, we drew on Bronfenbrenner’s Socio-Ecological Model (SEM) (21), which conceptualizes individual behavior and wellbeing as shaped by nested, interacting levels of influence–from personal factors to interpersonal relationships, community contexts, and broader policy environments. This framework provided a useful lens for interpreting the multilevel nature of participants’ routine disruptions and adaptations. Our approach was further informed by Caperon et al. (22), who developed a socio-ecological model for community engagement in a health program targeting underserved urban populations. Their emphasis on integrating structural, social, and spatial considerations into public health programming complements our focus on routine change, social connection, and access to supportive environments.

Building on this theoretical foundation, we conducted reflexive thematic analysis following the six phase approach outlined by Braun et al. (23) and adapted from Finlay and Guzman’s analysis of earlier COVID-19 Copy Study data (18). First, all authors familiarized themselves with the responses. Second, the team analyzed random subsets and generated initial codes, which informed the creation of a flexible codebook. Each author then coded a subset of responses in Dedoose. Regular meetings supported codebook refinement, peer debriefing, and discussions of emerging patterns and outliers. Third, we grouped codes into candidate themes. Fourth and fifth, we reviewed and defined each theme to ensure analytic clarity and alignment with our research question. Finally, we collaboratively drafted the results using quotes representative of our sample, which were lightly edited for grammar. To enhance methodological rigor, we employed strategies including author reflexivity, peer debriefing, and clear audit trail throughout analysis (24).

## Results

Over 1,300 participants responded to at least one of the three quantitative questions asking about altered routines (Table 2). The average age of participants was 71 years, and the majority identified as female (74%), Non-Hispanic White (93%), retired (65%), married or in a relationship (66%), highly educated (88% with at least a 4-year college degree), and residing in an urban community (91%) (Table 1). We analyzed over 2,500 unique open-ended qualitative responses, with 40% (*n* = 1,006) related to altered social activities, 33% (*n* = 844) related to exercise, and 27% (*n* = 693) related to creative or educational routines (Table 2). It should be noted that the overall sample underrepresents men, racial and ethnic minorities, individuals with lower educational attainment, and those living outside the state of Michigan.

**TLE 2:** Emotional health and routine change responses of qualitative respondents, COVID-19 coping study, United States, 2023 (*N* = 1,309).

Survey response	*n* (%)
Has your exercise routine changed since the COVID-19 pandemic onset?	
No	460 (35.3)
Yes	844 (64.7)
Non-missing open-ended response relating to changes in exercise routine	
No	0 (0.0)
Yes	844 (64.7)
Has your social routine changed since the COVID-19 pandemic onset?	
No	291 (22.4)
Yes	1,011 (77.6)
Non-missing open-ended response relating to changes in social routine	
No	5 (0.4%)
Yes	1,006 (76.9)
Has your educational/creative activity routine changed since the COVID-19 pandemic onset?	
No	588 (45.4)
Yes	706 (54.6)
Non-missing open-ended response relating to changes in educational/creative activity routines	
No	13 (1.0)
Yes	693 (52.9)
Has depressive symptoms^†^	
No	1,033 (79.1)
Yes	273 (20.9)
Mean (SD) CES-D Score	1.4 (2.1)
Has anxiety symptoms^§^	
No	1,081 (83.8)
Yes	209 (16.2)
Mean (SD) beck anxiety score	7.4 (2.3)
Feels lonely some or most of the time^⧧^	
No	4.3 (1.7)
Yes	302 (23.4)
Mean (SD) UCLA loneliness score^⧧^	4.3 (1.7)
COVID-19 worry	
Not at all worried	271 (20.7)
Slightly worried	648 (49.5)
Somewhat worried	261 (19.9)
Moderately worried	110 (8.4)
Extremely worried	19 (1.5)

SD, standard deviation. All characteristics were measured in April/May 2023, except for sex, race/ethnicity, education, marital status, and region of residence, which was measured in April/May 2020. All measures are unweighted. ^†^Derived from the 8-item CES-D depression screener. ^§^Derived from the Beck Anxiety Inventory. ^⧧^Derived from the UCLA Loneliness Scale.

Regarding *how* routines had changed, approximately one-quarter of responses referenced doing more either in-person, online, and/or in general. Additionally, 2 in 5 responses identified participating in *less* routine activities overall. Very few responses (~6%) mentioned that their routines were either getting back to normal or were fully back to normal, while 1 in 5 responses referenced no longer engaging in routines that they had prior to the pandemic.

**Figure 1:**
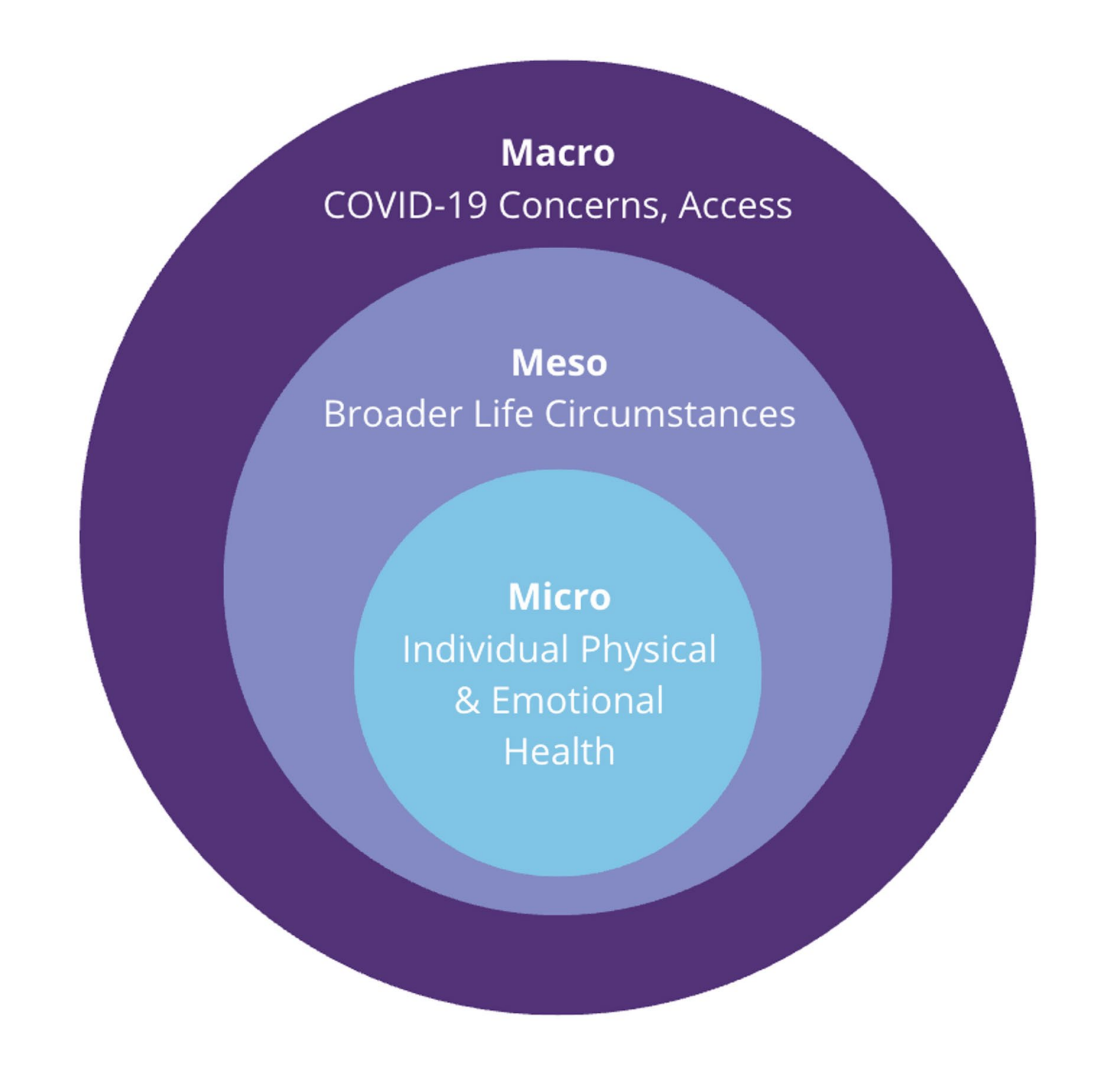
Multiscalar socio-ecological systems underlying routine changes among US older adults since the COVID-19 pandemic onset.

Using the Social-Ecological Model by Urik Bronfenbrenner as a guiding framework due to its emphasis on the way social and environmental factors impact one’s behavior, we identified five key themes in our analysis of *how* and *why* the routines of some aging Americans have continued to change 3 years after the onset of the COVID-19 pandemic. Reasons include: (1) COVID-19 exposure and risk, (2) altered access, (3) broader life circumstances, (4) emotional health, and (5) physical health (Figure 1; Table 3).

**Table 3:** Thematic framework of *how* and *why* routines have changed since the COVID-19 pandemic onset.

Level	Theme	Sub-theme
Macrosystem	COVID-19 exposure and risk	Avoid certain places
		Avoid untrusted people and groups
		Preference change
	Altered access	Increased access linked to the pandemic
		Decreased access linked to the pandemic
		Weather
Mesosystem	Broader life circumstance	Relocation
		Death
		Employment changes
Microsystem	Emotional health	Worsened emotional state
		Coping behaviors
		Disengagement and re-engagement
		New routines and peer influence
	Physical health	Physical limitations
		Aging

## COVID-19 exposure and risk

A large majority of participant responses referenced perceptions of COVID-19 exposure and risk as reasons underlying their altered routines. Many participants focused on strategically avoiding certain places and people and adjusting their routines to reduce exposure, navigating daily life in a complex landscape of COVID-specific anxiety.

### Avoid certain places

Many participant responses regarding COVID-19 exposure and risk noted continued avoidance of particular places given concerns about viral exposure. While some participants identified specific places or circumstances such as busy restaurants and theaters, many discussed these trends more generally. They now avoided going out in public altogether, a notable divergence from their pre-pandemic routines of more carefree engagement in public life.

Crowds and large group gatherings were of concern in many responses regarding place avoidance. Participants still felt uncomfortable in close proximity to others, with most attributing this reason to altered social routines. Participants reported a preference for smaller group sizes such as Jane (79F), who shared that she “rarely goes to movies or theaters or large group social gatherings. It feels too risky. I have been avoiding these since 2020 and have stayed much healthier in general.” Others indicated that they avoid leaving their perceived safe space at home altogether. This included Robin (77F): “I try to avoid contact with strangers because I am afraid of infection.”

Increased awareness of air circulation and the potential for viral airborne transmission also influenced participants’ place avoidance. When examining amenities avoided, a small subset of participants noted preferences for outdoor and well-ventilated areas to gather with others, with a majority attributing this to changing their social routines. Activities such as dining indoors at a busy restaurant, seeing a play at a crowded theater, or exercising at a fitness center in close proximity to others were frequently mentioned as both specific places to be avoided and reasons to avoid going out in public altogether. “We rarely go to indoor venues - so no libraries, coffee shops, galleries, etc.,” said Paula (62F). She continued: “We eliminated activities with strangers at indoor venues due to the risk of COVID.”

Participant attitudes toward COVID-19 precautions varied. For the small number of participants who avoided places due to a perception of excessive COVID-19 restrictions and public health guidelines, all identified as male. Wayne (68 M), for example, reported that he stopped attending educational classes because the instructors “demanded masking unreasonably…so we chose to stop [going to] those.” Conversely, others felt precautions were insufficient or were bothered by how they were followed. “I avoid dense crowds, and am very choosy about the locations we go to. If they had a history of non-compliance during COVID, we no longer go there. If they were compliant and still are, we go there,” wrote Dorothy (77F) about her change in social routine.

Moreover, the qualitative responses revealed a complex relationship between individuals’ levels of worry about COVID-19 and their place-avoidance behaviors. When asked to rate their worry about COVID-19 (Likert scale ranging from “Not at all worried” to “Extremely worried”), many participants who expressed “no worry” about COVID-19 did not align this sentiment with their reasons for avoiding certain places. For instance, Virginia (76F) reported that she is not at all worried about COVID-19. However, when prompted as to why her social routine had changed, she wrote, “I socialize less in person, especially when there is a large group of people, to minimize the chance of infection.”

### Avoid untrusted people and groups

Similar to the broad avoidance of going out in public, a smaller group of participants explained that their routines were altered because they avoid interacting with others *in general* due to concerns about COVID-19 exposure. Most participant responses attributed this to be a factor changing their social routines, with a minority attributing it to creative or educational and exercise routines. For some, this meant no longer engaging with friends or family as they did prior to the pandemic onset; for others, it meant not engaging with *anyone* they might encounter outside of the home. The degree to which participants described their avoidant behavior varied, with some describing routines altered to avoid people broadly and others only engaging with trusted individuals.

A major factor driving person-avoidance was whether or not someone wore a mask. Many responses that reported avoidance of people as a cause of routine change expressed the belief that those not wearing masks exhibited a general lack of care for the wellbeing of others. “I do not participate in any indoor activities (restaurants, movies, shopping, etc.) where there will be unmasked people, which greatly limits my social life,” wrote Vicki (59F). More broadly, there was a significant concern about inconsiderate actions in public, such as not wiping down exercise machines after use. Some indicated that they would actively avoid people who were not taking precautions or appeared to not take COVID-19 seriously. “I only engage with people that I know are following protocols,” shared Anita (69F). This behavior was rooted in a desire to stay away from individuals perceived as irresponsible or indifferent to the risks posed by the virus. This often led individuals to group those that were not taking COVID-19 seriously into a separate category than the rest of the population. For example, one participant assumed that younger folk were more likely to be inconsiderate in their actions than other age groups. Doris (84F) said, “I am not comfortable going to the gym since COVID as they are full of young people … who do not take any precautions.”

Political differences contributed to just under half of reported avoidance of people as a cause of routine change. Loss of respect for those not taking COVID-19 seriously was commonly cited, with many respondents delineating between those who were vaccinated and those who were not. “Even though I am fully vaxxed, I do not go to the movie theater, no bars or concerts,” wrote Richard (64M), “I do not want to be around those stupid people that will not adhere to changing their behaviors and believing in science and fact. Remember, this is all political.” This division often influenced social interactions and led to a pronounced avoidance of unvaccinated individuals, or those perceived as dismissive of the pandemic’s severity.

### Preference change

Participants’ reduced-risk activities included attending virtual gatherings, interacting in-person with smaller groups, going out during less busy times, being more selective in choosing activities, favoring outdoor settings, and practicing social distancing. This routine change most commonly affected social routines, but it should be noted that exercise and creative and educational routines were also altered by preference change for a minority of participants. Regardless of the type of routine changed or method of routine adjustment, many respondents altered their behavior around strangers, opting to limit contact or avoid engagement with unfamiliar people altogether. This cautious approach often informed their choice of activities or settings.

Participants who described engaging in reduced-risk activities typically did so in multiple areas, such as going out during less busy times *and* only participating in outdoor activities or being more selective *and* social distancing when engaging with others. Many of our participants with adjusted routines mentioned a preference for outdoor activities because social distancing was easier to maintain compared to indoors. Evelyn (77F), a Minnesota resident, reported being “more focused on being outside when possible. I believe that outdoors is our safest place.” Similarly, some participants were more selective about their social interactions. Participants spoke about weighing the risk of exposure if they participated in some activities, and many used this as a mechanism to decide whether to participate. For example, Theodore (67M) shared, “We do not just go out on the spur of the moment. I weigh the risks more now.” Some participants also reported a preference for virtual gatherings so they could avoid risking exposure altogether. “To avoid COVID,” Pearl (78F) wrote, “most of my relationships have shifted to online.” The use of Zoom and other online communication platforms were frequently adopted in place of in-person gatherings. Many felt that smaller groups were more manageable for their vulnerability or opted to go out during less busy times to minimize exposure to COVID-19. “I think more about safe times to go out where there are less people. I limit public exposure as much as possible,” Eileen (80F) wrote.

Interestingly, many respondents who preferred gathering in smaller groups reported feeling *no worry* about COVID-19. Monica (65F), for example, expressed that she did not feel worried about COVID-19. When prompted to discuss her routine change, however, she said, “I do not go shopping just for something to do,” highlighting a broader shift toward more purposeful outings. Overall, there was a pattern of decreased spontaneity and increased selectivity regarding social interactions and outings. Participants often limited ambient social contact by spending less time window shopping, engaging in casual conversations, and being around strangers as compared to before the pandemic.

While participants shared widespread routine changes, a small number also expressed having a boosted sense of safety, largely due to higher vaccination and lower infection rates. This increased sense of security encouraged these respondents to feel more comfortable going out in public and socializing, and therefore resume activities and feel more at ease in their daily routines. “I attend events and activities without thinking of COVID risk, unless I know there have been recent cases at a site,” expressed Colleen (64F), who lives in rural Wisconsin. In addition to personal vaccination, respondents noted that they felt safer because they only spent time with groups of people who were verified to be vaccinated, indicating a level of selectivity and trust in their social circles. Lillian (70F) wrote, “I did not socialize with strangers at the beginning of COVID… Now I trust my social group. They are all vaccinated, and we have trust in each other.”

## Altered access

Some responses indicated that altered daily routines were intrinsically linked to accessibility. Comparisons between current and pre-pandemic routines highlighted how the COVID-19 pandemic both increased and decreased access, influencing opportunities for daily routine activities. Participation in social, exercise, and creative and educational routines also differed depending on weather conditions.

### Increased access linked to the pandemic

Virtual activities increased availability for some participants, who were on average younger than the rest of the study cohort. For some participants, such as Vou’gul (63F) who identifies as American Indian or Alaska Native and lives in rural California, activities that were previously inaccessible due to their residential location are now possible:

I've participated in a number of online workshops and classes, which really weren’t available as much before COVID. I might even participate in more online classes than in-person, partly because the in-person classes/workshops that I'm interested in aren't as readily available where I live.

A minority of participants who reported social routine changes noticed that the pandemic-induced switch to online communication enabled renewed and sustained connections with people who live in different geographic regions. Gloria (81F), who lives in the urban Midwest, shared:

My siblings and I zoom regularly, which we never did before COVID. Since we live all over the world, it's a wonderful addition. The same is true with some groups of friends who live in other places.

The option to participate virtually also encouraged participation while navigating sickness and mobility limitations. Gail (61F) shared:

We use Zoom a lot! Which I love. My small movie group now meets more often at a friend's home or chats on Zoom. Changed first because of COVID, but some are getting older adult and less mobile.

Others appreciated the convenience of staying at home and not having to worry about traffic or parking; finding remote options was an efficient way to save time and money. Donna (63F), for example, expressed: “I did very little online before the pandemic. Now I do so entirely online. It is much cheaper and a more efficient use of my time.”

### Decreased access linked to the pandemic

Similarly, a minority of responses indicated a decline in access. Individuals reporting decreased access tended to be older compared to the rest of the study sample.

These participants shared that many venues offering in-person activities before the pandemic switched exclusively to virtual events. While the majority reported benefits of remote options, a small number felt that virtual options reduced or diminished their abilities to participate in events and activities. Shirley (65F) wrote: “Many things went online and I have not gone back.” Another participant, Ila (63F), Another participant, Ila (63F) wrote, “Routines are less in-person, way more Zoom. And, overall, fewer opportunities than before … it’s sad.”

Participants reported a decrease or loss of access to places they frequented before the pandemic. The most commonly cited reason for this decrease or loss was a change in recreational memberships, with a majority of responses referencing their exercise routines. As aforementioned, concerns about exposure to the virus were one of the drivers behind these changes, specifically in discontinuing memberships to exercise facilities. Scott (59 M), from a rural Midwestern community, shared:

I had been a member of the YMCA for decades, but quit in the pandemic because of poor ventilation and crowded quarters in the weight room. It made me sad because I miss the community, even if it was casual and occasional.

Although some participants changed their memberships due to fear of exposure, others realized that they prefer alternatives. Judy (74F) wrote: “Prior to the pandemic, I belonged to a gym and had a personal trainer. Now, I exercise at home or outdoors in secluded areas. This is not because I fear the pandemic, but because I enjoy the solitude.” Similarly, Elsie (63F) noted:

I initially changed because the gym I had been going to was closed for an extended time. I discovered an online alternative to the classes I had been taking at the gym which I much prefer and have never felt the need to return to the gym since then.

Participants also expressed deep connections with recreational facilities in their neighborhoods. However, permanent closures or facility relocations caused many to alter or withdraw participation in related activities altogether, resulting in emotional disconnection and reduced engagement. Ann (76F) shared:

For almost 10 years, I had been working out several times a week at a gym that I absolutely loved. Everything about that place made me happy. The gym was a victim of the pandemic and had to close permanently. I started working out at home via Facebook Messenger. But, it isn't the same. I miss the camaraderie so much. And I don't want to join a big gym--too many people, too many young people (often too casual about infectious diseases)–so that very happy part of my life is now over.

Similarly, Camille (67F) recalled:

Since we retired in May 2020, our exercise routine did change to include activities around our home … I was really disappointed when the hot yoga studio closed during the first couple months of the pandemic and never reopened. It has been difficult to replace that kind of activity since then.

### Weather

Participants expressed increased motivation to engage in physical activity during nicer weather, hinting at a preference for outdoor activities. Brenda (60F) mentioned: “I used to go to a gym, but now I just walk a lot. I have no interest in going back to a gym. I’ll swim once the weather is warmer, but I will not swim in an indoor pool.” Katherine (75F), also noted:

Our Aqua Aerobics classes moved to the outdoor pool, which I'm really enjoying (though it has been cold this winter). I've found an outdoor Zumba class which I've been enjoying, but it's been canceled more frequently lately due to a very wet winter.

Others decided to purchase home exercise equipment during the pandemic which they use during inclement weather. This includes Christine (63F) who lives in the Midwest: “I bought a Peloton, and I use that regularly, especially in the winter when I’m unable to be outside due to ice and snow.”

Many participants who mentioned the impact of weather on their social activities noted that inclement weather combined with heightened awareness of the increased risk of COVID-19 transmission in indoor spaces hindered their participation. Elizabeth (72F) is still slightly worried about the virus, shared:

My husband and I are hoping to dine out more as the weather warms and outdoor seating becomes available. We still do not feel comfortable being packed into places and maybe never will.

Additionally, Maya (64F) who lives in a harsher Midwestern climate, wrote:

I used to go to a lot of concerts and comedy clubs. Now I don't. I'm no longer comfortable in crowded spaces so I don't do that anymore. I still prefer outdoor socializing, so that's limiting especially in the winter.

## Broader life circumstances

Many responses emphasized broader life circumstances that overlapped with the pandemic as shaping their routine changes. These included residential relocation, the death of a loved one, and changes in employment status.

### Relocation

Relocating since the pandemic onset was one of the most commonly mentioned life events. Many participants described how moving altered their social routines, most often noting decreased socialization due to disrupted community ties or challenges forming new ones. For example, Sue (68F) in rural Pennsylvania reflected on her experience: “We socialize with fewer people but it’s also a factor of moving. Many pre-COVID activities did not restart, making it harder to meet new people.”

Still, others described improved social opportunities after moving. Many of those who discussed relocation noted positive changes in their routines, often due to a more socially connected environment. Vita (60F), for instance, shared: “I made new friends when I moved, and now I socialize more.”

Participants who moved to retirement or assisted-living communities frequently described greater access to organized social activities. These types of environments often offered built-in opportunities for engagement, which some participants found particularly beneficial. Edna (72F) explained:

We moved to a 55 plus community that has a very active social calendar. We had become quite isolated in [our previous residence] because of my wife's stroke/cognitive impairment and this allows me to be active and involved near home. I've made several friends and am involved in several activities.

Relocation also affected access to services and amenities that shaped daily routines. Some participants reported diminished access to natural amenities in their new environment, such as nature or fitness-friendly environments, while others noted improvements. Leonard (74M), who moved across the country, described a change in his exercise routine: “My exercise routine is different primarily because my wife and I moved from Colorado, where I hiked in the foothills for cardiac/aerobic activity, to Florida, where I struggle to substitute something as effective and enjoyable.”

Creative and educational activities were also impacted by relocation. For some, opportunities declined in their new setting; for others, new resources became available. Robert (67M) described his experience adjusting to both work-from-home and a new environment:

A combination of adjusting to work-from-home and moving … caused all kinds of changes. I play piano more than I did. I read somewhat more. I don't go to theater or concerts like we did in [our prior residence] … We used to have concerts and other activities that we knew about and could invite others to; we're still learning what's available where we are.

Others, like Diane (59F), reported enhanced opportunities for outdoor activity after relocating to a warmer climate: “I actually exercise more now because I moved to a warmer climate and I can get outside more, regardless of COVID.”

### Death

A small number of participants described how the death of a spouse, family member, close friend, or pet had deeply affected their routines. These responses most often referenced reduced social engagement and diminished physical activity, along with heightened emotional distress. Participants who experienced bereavement often described feeling lonely, withdrawn, or less motivated to maintain prior routines. Alice (72F) shared: “I watch way more TV/videos than before the pandemic, and since my husband’s death, it’s pretty much all I do.”

While most participants who mentioned death described loss and disruption, one participant also noted new possibilities emerging from her changed circumstances. Margaret (85F) described:

Transferring from being a caregiver in a house to a widow living alone in a senior community has been totally life changing for me. I'm in a new phase of my life but it had nothing to do with COVID. I'm still grieving the loss of my husband after 63 years of marriage but I'm basically a happy person and I have wonderful support from my large extended family.

It should be noted that like many of our other participants discussing caregiving, Margaret discussed her experience with caregiving largely in the context of age-related illnesses rather than COVID-19. Across these narratives on bereavement, grief was interwoven with broader reflections on social connection, daily structure, and wellbeing.

### Employment changes

Participants also discussed how changes in employment, including retirement and the shift to remote work, had reshaped their routines. For some, the onset of the COVID-19 pandemic overlapped closely with the transition into retirement, making it difficult to disentangle changes due to life stage from those driven by the pandemic. Nonetheless, several participants pointed to ways the pandemic altered or exacerbated their post-retirement experiences, such as reducing opportunities for social connection or reshaping how they structured their time.

Many newly-retired individuals reported fewer social opportunities and increased time spent at home, especially if their social lives had previously been connected to the workplace. Beverly (64F) shared, “I was working before the pandemic and had many friends. Now that I retired, my world has shrunk to the people who live in my building and a few others.” Similarly, some participants described loneliness and isolation after transitioning to remote work. Leslie (64F) noted: “Less socializing at work - many folks have not come back. Luckily, I had a social network before, it would be really hard if I did not. It’s still not the same as it was before and it can be lonely.” Others described how retirement allowed more time for creative engagement or social connection. A few individuals reported intentional reshaping of their social worlds post-retirement. Betty (71F), for example, reflected:

I retired shortly before the pandemic started, and much of my socializing was profession-related. The pandemic gave me a wonderful pause to seriously consider how I wanted to engage in this next chapter of my life. I tend to be a social individual, so I am pleased that I have developed a “post-retirement tribe” (that certainly includes previous colleagues) to feel socially fulfilled.

Exercise routines were another domain where newly-retired participants and those who switched to remote work during the pandemic described changes, most often improvements. Many replaced time previously spent commuting with physical activity. For example, Frank (67M) began walking during the initial stages of the pandemic and has continued since:

I walk a lot more. This basically happened during COVID. I stopped going into the office, which saved me about 1.5 hours per day in commute time. More or less out of boredom I started walking on paths in a nearby park. Now it is an unusual day when I do not walk at least 3 miles.

Similarly, Martha (67F), who had retired but still worked part time remotely, noted, “I now have the flexibility to go take classes at the gym 4 or 5 days a week. I enjoy the exercise, socializing with other members, and the routine!” Joanne (63F) also retired and worked part-time, which she enjoyed very much because “I now have more time for yoga and walking than when I worked full-time. Loving it!” However, not all transitions improved activity levels. Some participants described diminished exercise routines because they no longer engaged in active transit like walking to work. As Larry (67M) explained: “Much less exercise than before COVID - mostly due to change in work (I used to walk and climb stairs at work as a part of my job).”

These reflections underscore how the pandemic not only co-occurred with but also shaped participants’ retirement transitions. While some changes may have unfolded regardless, COVID-19 directly influenced how individuals adapted their routines, particularly in terms of physical activity and access to social opportunities.

## Emotional health

Many participants described how their emotional health shaped or was shaped by changes to their daily routines compared to before the COVID-19 pandemic. While some described how the disruption led to increased stress, anxiety, or loneliness, others shared how these changes ultimately helped them build new habits or reevaluate how they spent their time.

### Worsened emotional state

Many participants reflected on how the pandemic heightened feelings of loneliness and worsened symptoms of depression and anxiety, which in turn disrupted their daily routines. This was especially common among individuals who had lower education compared to the overall sample or were single. Susan (57F) who is divorced, explained: “I rarely go out to socialize since COVID. I have anxiety, depression, and fibromyalgia and my need to socialize has diminished. I have at times isolated myself without even realizing it until I really pay attention.” Some shared how their mental health was impacted when their own COVID-19 precautions did not align with those of friends or family, straining relationships and deepening emotional distress. Teresa (64F) remained extremely cautious, said:

After lockdown, I tried socializing in-person with friends and family. But they were engaging in risky behavior that I wasn’t comfortable with. They were also very negative and even abusive, which gave me another reason to avoid them … It made me very anxious and depressed.

### Coping behaviors

Others described how emotional strain prompted them to build new routines to help them cope, often through exercise or being more selective in how they spend their time. Participants who shared these stories often described themselves as less anxious or lonely than others and were more likely to be retired and/or highly educated. Nancy (67F) started a daily walking routine with her husband during the pandemic to help with stress. This has continued ever since: “We walk every day and have since I was sent home from work in March 2020. At first it was a way to deal with the stress of the pandemic and then we just made it a habit.” Maria (69F) shared a similar story: shared a similar story:

I started walking before the pandemic but not consistently or for very long. With the pandemic, it became my coping mechanism and I started walking at least twice a day and gradually increased my distance. I now walk 4–5 miles a day at least 5 days a week.

While some routines emerged out of necessity, they often became intentional lifestyle shifts. Participants shared that they no longer felt obligated to socialize or fill their calendars and instead embraced a slower pace. This included Debra (68F), who wrote:

We go out less with others because either we are more cautious or they are more cautious. Also, after getting out of the pattern of going out all the time to socialize, we discovered we enjoy time at home without the hectic pace of life we had pre-COVID.

Sandra (80F) echoed this shift:

I had been pretty much a full-time volunteer (even more than full-time) before the pandemic. I went to evening meetings at least 3 or 4 nights a week, plus some daytime meetings. [The pandemic] made it much easier to say “NO.” Now, I'm down to just three groups that I meet with on a monthly basis, where much of my role can be done at home, on a phone or computer.

Other participants described a new sense of self-awareness about their time, energy, and creative expression. James (69M) wrote:

My exercise program is less social. My yoga and Pilates mat classes are done via Zoom. I teach fewer in-person dance classes. I retired from full-time work, in part, because of the challenges of COVID and what COVID revealed to me about the value of my personal satisfaction.

In choosing to forego prior obligations, some participants used the time to explore more creative opportunities at home. Bonnie (77F), who has retired, reflected on how she used to frequent concerts, lectures, and community events:

Since the pandemic, I've stopped doing those things, because they were the events I consider to have the highest risk of exposure to the virus, and were the things I could most easily pass on participating in … I have a rich creative environment at home, so I'm not starving for creative or educational stimulation elsewhere.

### Disengagement and re-engagement

Some participants reflected on how early disruptions to their routines lingered long after the initial lockdowns. For many, the structure of their day-to-day life had not returned to how it was before the pandemic. Mary (71F) noted: “My routine became non-existent and I am trying to rebuild it.” Participants such as Kathleen (61F), noticed: “We have fewer visitations with friends. We just have not gotten back into our previous routine. I guess we got out of the habit.”

Others echoed this ongoing disconnect, describing how their habits shifted toward inactivity or home-based routines. “I was more social before. I changed due to inertia and the fact that my friends’ routines also changed,” shared Denise (62F). A habit of staying home typically had the greatest impact on individuals’ social routines. Participants who described a habit of staying home since the pandemic onset typically mentioned spending less time socializing, such as Sally (73F): “I go out much less than I did prior to the pandemic. We have gotten in the habit of staying home much more of the time instead of socializing with family and friends.” For some, the closure of spaces like gyms made it harder to maintain previous exercise routines. Roger (74M) shared: “I used a public gym prior to Covid-19. The pandemic broke that pattern which I’ve not resumed.” While many participants expressed frustration about the difficulty of returning to pre-pandemic routines, others were optimistic or had already begun re-engaging. Jerry (76M) wrote: “My wife and I quit attending indoor events or activities, and even crowded outdoor ones, at the start of the pandemic and have not yet returned, though we are thinking about it.” Joyce (79F) shared: “One major change is that I used to sit in on university classes (as a retiree). I stopped doing that. I think I’m ready to take it up again.” Some even noted they were waiting for their community to reopen before resuming their pre-pandemic routines. Kelvin (67M) expressed: “I stopped my 2–3 times a week at the gym on March 1, 2020 and plan to resume when a new gym opens in our town soon.”

Some participants mentioned that their routines were already beginning to go back to normal and were even picking up some new hobbies along the way. Christina (63F) expressed: “Socializing is still just a bit less frequent, but will probably be back to “normal” soon. Even before COVID, most socializing was at someone’s home or outdoors, like camping. That routine was changed to reduce exposure risk.” Similarly, Rose (69F), a Midwestern retired woman wrote that her social routine is “less frequent but that continues to change for the better.” Participants also noted small personal goals they were working toward, such as Barbara (63F): “I am trying to exercise more regularly.” Lori (63F) shared: “I decided to take a community college class which has been a tremendous help. Hope I can do this again.”

### New routines and peer influence

Some participants described how the pandemic-induced changes opened space for new hobbies and a deeper appreciation for daily life. Many said they felt more connected to their interests or relationships than before. Lisa (69F), a retiree from the Midwest, used the pandemic as a means of encouragement to try new things. She shared, “I am doing more creative endeavors than before COVID. Not sure why. Because it’s fun!.” Carl (78M), who was also retired, started expanding his creative routine: “I did participate in a musical for 15 performances as a part of the ensemble. It was so much fun!.” Dawn, who joined a community camera club, reflected: who joined a community camera club, reflected: “This gets me out of the house to do something I love. I felt it was important to not be cut off after retirement and it brings me a lot of joy.” Julie (62F) shared how she returned to group activities: “I have joined a fitness club and attend group classes. I wanted to be more social again and wanted the structure of an exercise class in addition to my solo biking, hiking, walking, etc.”

Participants also found creative workarounds to replace routines they had lost. Peggy (77F), whose exercise classes were canceled, found a new way to stay active: “Now I walk outside or I take my dog in bad weather to Lowe’s-they let us walk our dogs in-store. One loop up and down each aisle is 1 1/2 miles.” Patricia (85F), who resided alone on the Northeast coast, used to swim frequently at the local pool in the community. After it shut down, she decided to replace it with other activities in her area. “I live 30 min from a seashore and boardwalk so I am gratefully able to take advantage of those activities instead!”

Once activities moved online after the shutdown, some participants with internet access realized that the online shift made it easier to pursue their routines. Ralph (72M) recalled:

We went to Zoom where our fitness instructor from the local Senior Center moved his class and have been able to keep up a more rigorous routine (3x per week) than previously (1x per week). It's been a great way to stay connected with the class, even meeting some new folks.

As aforementioned about altered access, the drastic shift from in-person communication to online allowed participants to socialize with friends and family in ways that they never had before. Janice (66F) noted: “During the pandemic, we started up regular Zoom calls with faraway friends that we had not kept in touch with as much. These are good things.” Responses also noted that the pandemic influenced participants to engage more often with like-minded individuals. George (76M) shared:

I have strengthened bonds with five men who are fellow cyclists. We ride in spring, summer, and fall, and meet for coffee in colder weather. This group is very significant for me. I have also maintained monthly social connections with another group of men.

## Physical health

A minority of responses discussed considerations of physical health, related or unrelated to the COVID-19 virus, in relation to routine changes. Notably, the majority of these responses focused on exercise routines, while discussion of aging related to physical health often mentioned social routines.

### Physical limitations

A small sample of participants mentioned COVID-specific symptoms or how the virus itself impacted their daily routines. Some briefly mentioned if they had COVID-19 at the time of taking the survey or if they had once been diagnosed with it, but generally provided little further detail. “I have had to cut back dramatically the amount and frequency of exercise due to physical symptoms since acquiring COVID,” Gerard (71M) wrote. Others referred to Long COVID symptoms but some, such as Harvey (90M), did not explicitly label it as such: “Since having COVID, I cannot exercise as much per day because of fatigue and being out of breath.” Other participants described themselves or a family member as immunocompromised and expressed concern for COVID-19 exposure, reflecting aforementioned isolation behaviors and limiting social routines. Louise (59F) wrote:

Obviously I changed my routine when COVID started. We have never gotten it back, partly because I am still worried about COVID. We have family members who were diagnosed last week, it is not gone and I am still immunocompromised … We talk on the phone when we can, that is about it.

Non-COVID-related physical health issues played a more significant role in routine changes among participants. Many cited various physical challenges such as hip and knee replacements, injuries, joint pain, fatigue, and other health conditions as reasons for altering their routines. Kent (93M) shared, “My wife and I have begun to experience physical impairments, she has arthritis and I have neuropathy. We both use walkers at home. Outside activity has been greatly reduced. Our family members assist with transportation if needed.” Physical therapy and rehabilitation were also mentioned by participants as influencing their daily activities, with many expressing a hope to get back to their old routines. “I’m not doing my regular exercise routine,” wrote Jackie (69F), describing her recent knee replacement, “so I am slowly rehabbing from that.” Additionally, a small number of those discussing non-COVID physical health indicated a proactive approach to improving their health through weight management, better eating habits, and increased exercise after the pandemic onset. “I exercise more often now,” Lloyd (65 M) explained. “Being older and overweight put me at higher risk for COVID-19, and now I focus more on living a healthy lifestyle, losing weight, and exercising.” This awareness of increased risk for contracting the virus was prevalent among participants, more so than actual descriptions of physical symptoms of or experiences with contracting COVID-19.

### Aging

For the minority of participants who mentioned it directly, aging itself was a significant factor in the adjustment of routines. There was a general sentiment of increased risk and vulnerability to COVID-19, which was attributed to advancing age. Amy (62F) explained her reduction in public outings, saying she was “not sure if COVID is done with us, but soon we’ll be considered higher risk due to our age.” Many responses discussing age came from men; this subset was, on average, older than the full sample, and nearly half of respondents in this group were over the age of 80. A majority of those who discussed aging felt that the challenges associated with growing older had a more profound impact on their lives than COVID-19 itself. “My reduced exercise routine does not have anything to do with the pandemic,” Julia (67F) explained. “It really just has to do with my age and pain levels.”

## Discussion

More than 3 years after the COVID-19 pandemic onset, the daily routines of many older adults are different, with potentially long-term implications for individual and public health. Our qualitative analysis aimed to identify *how* and *why* daily life remains different for physical, cognitive, and social health routines as we enter an endemic phase of COVID-19 (15). Participants reflected on nuanced and unforeseen changes to their routines, citing fears of viral exposure, multidimensional changes in access to services and amenities, physical and emotional health concerns, and broader life circumstances overlapping the pandemic.

Concern about COVID-19 and other viral exposures was a prominent theme in participant responses. While some individuals had returned to pre-pandemic activity levels, many others noted persistent lifestyle changes to accommodate these newfound and exacerbated fears. While other studies have found differing levels of viral exposure concerns between urban and rural settings (25), many of our study’s rural participants echoed similar feelings about the pandemic to those living in urban areas. There was also a dichotomy between how participants responded to COVID-19 precautions: some thought public guidelines and individual practices (such as masking) were too stringent, while others thought the opposite. Although responses regarding COVID-19 precautions were divided–some believing public guidelines were too stringent while others thought otherwise–the presence of precautions played a significant role in influencing place-specific avoidance. We also found a prominent disconnect between worry about COVID-19 and changes to routine–although individuals were not as worried about the COVID-19 virus, their routines are still different 3 years after the pandemic. Such a relationship can be explained by Ajzen’s theory of planned behavior (26), which emphasizes how subjective norms, perceived behavioral control, and one’s attitude toward behaviors can all influence their engagement in certain behaviors. Therefore, individuals may report little to no worry about the COVID-19 virus, yet still have a different routine 3 years after the pandemic due to the influence of others close to them (subjective norm), things out of their control such as closures and accessibility (perceived behavioral control), or nuanced perceptions of the importance of the behavior in their lifestyle following the pandemic (attitude toward behavior).

Our study extends the timeframe of previous studies documenting avoidance of people and disengagement from group-settings in the early acute pandemic phase (7). Nearly a quarter of responses indicated a continuation of avoidance more than 3 years after the pandemic onset. The degree to which participants described avoidant behavior varied, with some describing routines altered to avoid people broadly and others only engaging with trusted individuals. Participants across diverse backgrounds and regions exhibited similar behaviors, suggesting that the impact of COVID-19 on social routines transcends communities across the US. This finding diverges from much of the literature, which highlights more pronounced impacts of the pandemic on rural (e.g., due to limited healthcare access and infrastructure) and urban communities (e.g., due to higher population density and exposure risk) (27, 28). Our results suggest that, while these structural differences influenced acute pandemic experiences, the longer-term persistence of avoidant behaviors may reflect more universal concerns, such as trust, perceived risk, and shifting social norms, rather than geographic or community-specific factors. Continuation of avoidant behavior disrupts essential health behaviors such as physical activity and access to social support. It also risks weakening both bonding and bridging social ties, which are critical for maintaining wellbeing and community resilience (5, 13). Bonding ties—close connections with family and friends—are essential for providing emotional support, while bridging ties—connections with broader network—facilitate access to diverse resources and opportunities (29). Reduced social connections have been linked to adverse health outcomes, including increased risk of mortality, cardiovascular disease, and cognitive decline (30). Strengthening these ties is vital for promoting longevity and mitigating the long-term health impacts of social isolation exacerbated by the pandemic.

There is a need to explore underlying psychological components, such as how factors like anxiety and pandemic-related trauma contribute to long-term shifts in behavior. Participants were less comfortable in the company of others, particularly strangers, reflecting a potential broader trend of prioritizing health and safety over social spontaneity. While these behavioral changes were established early in the pandemic (31), their persistence more than 3 years later suggests a lasting shift in social interaction norms among older adults. This has potential implications for public health and community planning. New approaches to building health-supportive, age-friendly communities could help mitigate risks of loneliness, reduced physical activity, and cognitive decline by fostering opportunities for safe social engagement, physical activity, and cognitive stimulation (6). These strategies might include designing accessible public spaces, promoting hybrid social programs that combine in-person and online participation, and increasing access to community resources tailored to older adults’ needs.

Participants also expressed how sudden, pandemic-induced changes to daily routines impacted their emotional health, with many reporting heightened feelings and experiences of isolation, loneliness, anxiety, and depression, especially due to adapting their routines to follow social distancing and stay-at-home orders. These individuals were more often divorced or single, and had more worry about COVID-19. A prior longitudinal study also using data from the COVID-19 Coping Study highlighted similar findings, with individuals who reported disruptions in their identity since the pandemic having higher symptoms of loneliness, depression, and anxiety at baseline, with no significant changes for depression overtime, but declines found for anxiety and increases in loneliness 1 year after the pandemic onset (32). Another study found symptoms of depression and anxiety were greater among individuals who voluntarily followed stay-at-home orders compared to those who did not (23). Even though COVID-19 restrictions have subsided, several participants continue to remain at home by choice, while others are constrained by limitations in access to online alternatives or the closure of facilities they relied on for their engagements. These changes had potentially detrimental effects: gym closures reduced physical activity, social distancing decreased the desire to socialize, and interactions with friends shifted in frequency and location. These participants reported greater feelings of loneliness and concern about COVID-19, highlighting the importance of maintaining daily routines (5, 33).

However, we also found that abrupt changes to routines *positively* impacted some older adults’ emotional health, more often those who were retired and highly educated. Many reported incorporating new routines or expanding previous ones to cope with the pandemic’s restrictions. Those with internet access adapted by shifting their routines online–often through video calls or social media, a benefit observed in other studies as well (2, 34). These participants reported little to no symptoms of loneliness. Given that loneliness has been associated with increased mortality risk among middle-aged and older adults (35), increasing online access could be a crucial strategy to reduce loneliness and its adverse health effects in this population (36). Further, some participants described routine changes that brought joy and newfound contentment with their lives, echoing findings from established research (37). Overall, these results underscore that adaptive routine changes positively impacted older adults’ emotional health and wellbeing. Our findings on adaptive routine changes align with prior research highlighting the effect of resilience and psychological flexibility on wellbeing during adversity. Older adults who demonstrate resilience, such as through proactive coping or acceptance, are more likely to maintain emotional stability and manage disruptions; these positive adaptations not only mitigate stress but can enhance perceived quality of life in the context of uncertainty or social isolation (38, 39).

While over one in 10 participants discussed physical health changes, only a small subset specifically mentioned COVID-19. Among those affected by the virus, some described long-term symptoms, including fatigue and breathlessness, that have reshaped their physical activity routines—an experience aligning with existing literature on Long COVID (40). However, most participants noted that aging and non-COVID physical health issues, such as chronic conditions, joint pain, or hip replacements, had a more significant impact on daily routines than the virus itself. These age-related challenges were seen as more influential drivers of routine changes due to the cumulative effects of physical vulnerability with age. This perspective is consistent with studies in other aging and older adult cohorts, which highlights how increased physical vulnerability and risk perceptions shape older adults’ daily activities (41). These findings underscore the need for interventions promoting physical health among older adults to address both age-related physical challenges and ongoing COVID-19 concerns, ensuring programs are adaptable to varying health needs.

## Limitations

There are notable limitations to our study. Since the survey was administered online, our sample only captures individuals who are digitally literate and have access to the internet. This could lead to a more biased sample, since older individuals who are lower educated, economically disadvantaged, and/or are living in a rural community are less likely to have the means to participate. The overall sample underrepresents men, racial and ethnic minorities, individuals with lower educational attainment, and those living outside Michigan. Future work in this area should investigate the disproportionate intersectional impacts of the COVID-19 pandemic on gender, sexual, racial, and ethnic minorities, as well as differential effects depending on economic cognitive health status. Long-answer responses varied in depth, from a few words to several sentences. While we asked participants about routine changes since the pandemic, the exact timing of these changes is unclear due to the cross-sectional nature of this study. Additionally, our survey questions required participants to reflect on their lifestyle prior to the pandemic, increasing the chance of recall bias. Trends by place were also limited, as zip codes were collected only at baseline, and some participants had moved. Moreover, given the multi-year span of the study, selective dropout may have occurred–participants with poorer health, those more socially marginalized, or individuals holding more politically conservative views may have been less likely to participate in follow-ups, potentially shaping the types of routine changes described. While our sampling may not be fully representative of older adults in the US, our results offer valuable insights for policy and best practices to support older adults’ social, exercise, creative and educational routines during future times of societal trauma and crisis. Longitudinal studies may further illuminate pandemic-related routine changes.

## Implications

Daily routines are crucial for physical, mental, and cognitive health. Our findings show that older adults continue to face routine disruptions years after the onset of COVID-19 and that these disruptions significantly impact vulnerable subpopulations, including those with limited mobility, chronic conditions, or compromised immune systems (18). Other affected groups include individuals who are single, newly retired, living in harsher climates, or have recently changed their residence. These individuals may struggle to maintain social connections and engage in physical activity or educational activities due to ongoing concerns about exposure and accessibility.

The pandemic has intensified spatial and social inequalities (42), necessitating further research to understand the lasting behavioral changes that can inform future public health policies. Our findings point to the importance of emotional health, social infrastructure, and daily routines as interconnected and underacknowledged components of resilience (38, 39), with implications for both individual recovery and community-wide crisis response.

Following the framework of the SEM (21, 22), our findings reinforce calls for multilevel strategies that account for individual needs, interpersonal relationships, and community infrastructure. As Caperon et al. (22) note, community engagement and crisis recovery are most effective when structural and social factors are addressed in tandem. Policymakers should prioritize the wellbeing of older adults by fostering environments that support bonding and bridging ties essential for both individual and collective health (29, 30). Promoting outdoor and well-ventilated activities and expanding online or hybrid engagement options will enable older adults to stay connected with their communities while reducing health risks. Additional strategies could include subsidized online and in-person exercise or artistic classes, tech literacy outreach programs with subsidized high-speed internet access, and sponsoring community events and social meetups in public spaces or private “third places.” Individuals should maintain routines and connectedness; however, it is essential that these micro-efforts are bolstered by community-and municipal-level public health efforts to emphasize the importance of engagement. These measures are vital for fostering emotional and social resilience, building community ties, and enhancing quality of life, especially in times of crisis (5).

Creating accessible spaces for safe socialization aligns with radical community care practices (43, 44), countering the individualization of health responsibility. By providing age-inclusive social spaces with lower risk of viral exposure, policymakers can help prevent further isolation among older adults, strengthening both individual and community resilience. This approach addresses immediate health needs and acknowledges ongoing social and spatial inequalities that impact older adults (42).

A commitment to inclusive public health policies will ensure older adults feel secure and valued in their communities, promoting both their social and emotional health in future crises. Public health efforts should enhance local recreational areas and create age-friendly spaces to encourage physical activity. Offering health promotion programs, social clubs, and creative or educational courses in multiple formats (online, in-person, hybrid) can help prevent social isolation and inactivity. By adopting these measures, policymakers can mitigate the long-term effects of routine disruptions and support the wellbeing of older adults as COVID-19 becomes endemic.

Finally, this study underscores the value of qualitative research in capturing the lived experiences of crisis recovery–insights that are often missed in large-scale quantitative surveys. Our findings reflect a wide range of emotional, social, and behavioral adaptations to prolonged uncertainty. As such, they offer critical guidance for designing compassionate, inclusive, and emotionally attuned public health responses to future disruptions.

## Data Availability

Data are available upon request to the corresponding author. Materials are available at https://www.covid19copingstudy.com/. The preregistration for the quantitative analyses conducted in this study is available at https://osf.io/kpbty/?view_only=46e85c0b7d9646acb5b7cdf8e7310513.
