# Supplementary material for: “It just isn’t the same”: altered routines among older Americans three years after the COVID-19 pandemic onset
Source: Front Public Health. 2025 Jul 9;13:1573302. doi: 10.3389/fpubh.2025.1573302 (PMC12283981; doi:10.3389/fpubh.2025.1573302)
Supplement: Supplementary file 1 [file Table_1.DOCX]

**Supplementary Materials**

Appendix A: Long Answer Survey Prompts - Routine Changes Page 1

Appendix A: Long Answer Survey Prompts - Routine Changes

**Exercise Routine**

Is your current exercise routine different compared to before the start of the COVID-19 pandemic in March 2020 (e.g., where or when you exercise, with who, how often, what type of exercise)? [Yes; No] If yes, How is your exercise routine different? Why did you change your routine? [long answer box]

**Social Routine**

Is your current social routine different compared to before the start of the COVID-19 pandemic in March 2020 (e.g., where or when you socialize, with who, how often, what type of activity)? [Yes; No] if yes, How is your social routine different? Why did you change your routine? [long answer box]

**Creative/Educational Activity Routine**

Is your current routine in creative or educational activities different compared to before the start of the COVID-19 pandemic in March 2020 (e.g., where you go, with who, how often, what type of activity)? [Yes; No] If yes, How is your creative/educational activity routine different? Why did you change your routine? [long answer box]
